# Exendin-4 attenuates atherosclerosis progression via controlling hematopoietic stem/progenitor cell proliferation

**DOI:** 10.1093/jmcb/mjad014

**Published:** 2023-03-02

**Authors:** Cen Yan, Xiaojuan Ma, Sin Man Lam, Yuejie Zhang, Yu Cao, Yuan Dong, Li Su, Guanghou Shui, Yingmei Feng

**Affiliations:** Department of Science and Development, Beijing Youan hospital, Capital Medical University, Beijing 100069, China; Center of Basic Medical Research, Institute of Medical Innovation and Research, Peking University Third Hospital, Beijing 100191, China; State Key Laboratory of Molecular Developmental Biology, Institute of Genetics and Developmental Biology, Chinese Academy of Sciences, Beijing 100101, China; Department of Science and Development, Beijing Youan hospital, Capital Medical University, Beijing 100069, China; Department of Science and Development, Beijing Youan hospital, Capital Medical University, Beijing 100069, China; Department of Science and Development, Beijing Youan hospital, Capital Medical University, Beijing 100069, China; Neuroscience Research Institute, Peking University Center of Medical and Health Analysis, Peking University, Beijing 100191, China; State Key Laboratory of Molecular Developmental Biology, Institute of Genetics and Developmental Biology, Chinese Academy of Sciences, Beijing 100101, China; Department of Science and Development, Beijing Youan hospital, Capital Medical University, Beijing 100069, China

**Keywords:** glucagon-like peptide-1 receptor agonist, hematopoietic stem/progenitor cells, proliferation, metabolomics, atherosclerosis

## Abstract

Beyond glycemic control, applications of glucagon-like peptide-1 receptor (GLP-1r) agonists (GLP-1 RAs) inhibit inflammation and plaque development in murine atherosclerotic models. However, whether they modulate hematopoietic stem/progenitor cells (HSPCs) to prohibit skewed myelopoiesis in hypercholesteremia remains unknown. In this study, GLP-1r expression in fluorescence-activated cell sorting (FACS)-sorted wild-type HSPCs was determined by capillary western blotting. Bone marrow cells (BMCs) of wild-type or GLP-1r^−/−^ mice were transplanted into lethally irradiated low-density lipoprotein receptor deficient (LDLr^−/−^) recipients followed by high-fat diet (HFD) for chimerism analysis by FACS. In parallel, LDLr^−/−^ mice were placed on HFD for 6 weeks and then treated with saline or Exendin-4 (Ex-4) for another 6 weeks. HSPC frequency and cell cycle were analyzed by FACS, and intracellular metabolite levels were assessed by targeted metabolomics. The results demonstrated that HSPCs expressed GLP-1r and transplantation of GLP-1r^−/−^ BMCs resulted in skewed myelopoiesis in hypercholesterolemic LDLr^−/−^ recipients. *In vitro*, Ex-4 treatment of FACS-purified HSPCs suppressed cell expansion and granulocyte production induced by LDL. *In vivo*, Ex-4 treatment inhibited plaque progression, suppressed HSPC proliferation, and modified glycolytic and lipid metabolism in HSPCs of hypercholesteremic LDLr^−/−^ mice. In conclusion, Ex-4 could directly inhibit HSPC proliferation induced by hypercholesteremia.

## Introduction

Approximately, >30% of type-2 diabetic patients would develop cardiovascular disease, which accounts for half of the mortality in these patients (Brown et al., 2021). Among all the anti-diabetic drugs, glucagon-like peptide-1 receptor (GLP-1r) agonists (GLP-1 RAs) have been applied as the second line of glucose lowering therapy. Irrespective of glycemic controls, the results of randomized clinical trials have consistently shown the beneficial function of GLP-1 RAs in the prevention of the incidence of major adverse cardiovascular endpoints, stroke, and chronic kidney disease in type-2 diabetic patients (Yan et al., 2020; Brown et al., 2021).

From pathological aspect, atherosclerosis is the underlying factor of cardiovascular disease. It begins with endothelial dysfunction, followed by low-density lipoprotein (LDL) retention in the sub-endothelium and inflammatory cell infiltration. In type-2 diabetic patients, treatment of liraglutide decreased circulating levels of inflammatory cytokines such as tumor necrosis factor-α, interleukin-1β (IL-1β), and IL-6 (Hogan et al., 2014). In line with that, administration of semaglutide, liraglutide, or Exendin-4 (Ex-4) has been shown to prohibit plaque progression in hypercholesterolemic mice via different mechanisms. For instance, nanoString analysis of the RNA extracted from plaque identified the decreased gene expression related to leukocyte recruitment to the lesion in apoE^−/−^ and LDL receptor deficient (LDLr^−/−^) mice on western diet. Ex-4 treatment promoted M2 polarization (Wang et al., 2017), resulting in reduced inflammation and retarded atherosclerosis progression (Tashiro et al., 2014; Wang et al., 2014a; Rakipovski et al., 2018). despite the anti-inflammatory properties of GLP-1 RAs on macrophages have been illustrated, whether and how they could modulate hematopoietic stem/progenitor cells (HSPCs) toward myeloid lineage production are not well defined.

HSPCs are the only cells that differentiate into all types of blood cells in one's life (Pouzolles et al., 2016). After birth, HSPCs reside in hypoxic bone marrow (BM) niche, and their homeostasis between self-renewal and differentiation is delicately regulated by intrinsic and extrinsic cues within the BM microenvironment. To date, accumulating evidence has indicated the intimate association between abnormal HSPC expansion, skewed myelopoiesis, and atherosclerotic progression in mice with diet-induced hypercholesterolemia (Gao et al., 2014; Sarrazy et al., 2016; Wang and Westerterp, 2020). In this study, the effects of Ex-4 on HSPC expansion, myeloid cell production, and plaque progression were investigated in LDLr^−/−^ mice on high-fat diet (HFD). Bone marrow cells (BMCs) of wild-type or GLP-1r^−/−^ mice were transplanted into irradiated LDLr^−/−^ recipients to assess HSPC function in BM reconstitution. Finally, targeted metabolomics was used to evaluate the metabolomes of HSPCs sorted from hypercholesterolemic LDLr^−/−^ mice treated with or without Ex-4.

## Results

### Ex-4 treatment attenuates plaque progression in hypercholesterolemic LDLr^−/−^ mice

**Figure 1:**
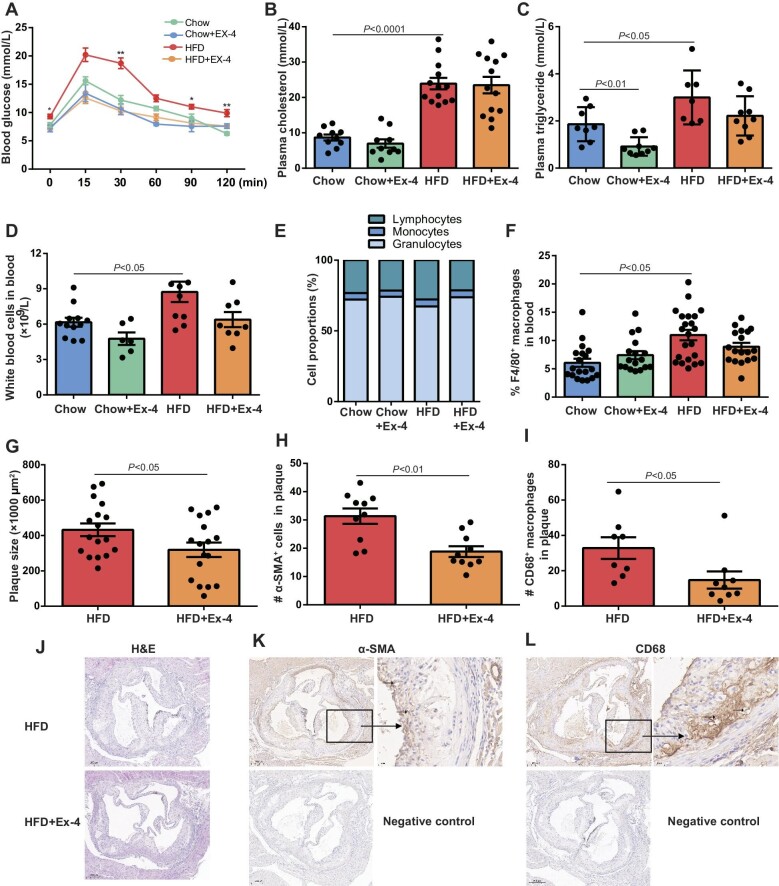
Ex-4 treatment attenuates atherosclerotic plaque progression in hypercholesterolemic LDLr^−/−^ mice. LDLr^−/−^ mice were placed on HFD for 12 weeks. After 6 weeks, mice received saline or Ex-4 treatment using minipump till the end of the experiment. (**A**) Glucose tolerance test. *n* = 4–18. *P < 0.05 and **P < 0.01 for HFD vs. HFD + Ex-4. (**B** and **C**) Plasma levels of cholesterol and triglyceride. *n* = 7–11. (**D** and **E**) Absolute and differential white blood cell count. *n* = 6–12. (**F**) White blood cells were stained for CD11b and F4/80 and analyzed by FACS. *n* = 18–22. (**G**–**L**) Histology and immunohistochemistry of plaque in the aorta. (**G**) H&E analysis of plaque size. *n* = 17 for each group. (**H** and **I**) The numbers of α-SMA^+^ and CD68^+^; cells in plaque. *n* = 8–10. (**J**–**L**) Representative images of H&E, α-SMA, and CD68 staining.

To explore the effect of Ex-4 treatment on atherosclerosis, LDLr^−/−^ mice were placed on HFD for 6 weeks and then embedded with minipump containing saline or Ex-4 for another 6 weeks. After 12 weeks on HFD, these mice were induced impaired glucose tolerance compared with those on chow diet (Figure 1A). Administration of Ex-4 reduced glucose levels in the entire time course of glucose tolerance test (Figure 1A). The area under the curve (AUC) was measured, and AUC values of glucose levels were 1251 for chow diet group, 1072 for chow diet with Ex-4 group, 1647 for HFD group, and 1108 for HFD with Ex-4 group, respectively. As expected, HFD resulted in a 2.5- and 1.6-fold increase in plasma cholesterol and triglyceride levels, respectively (Figure 1B and C). However, Ex-4 treatment did not alter cholesterol and triglyceride levels in LDLr^−/−^ mice on HFD (Figure 1B and C).

In line with previous reports (Feng et al., 2012; Seijkens et al., 2014), HFD promoted leukocytosis, myeloid cell production, and F4/80^+^ macrophage frequency (Figure 1D–F). Accordingly, plaque size was 1.4-fold lower in hyperlipidemic mice receiving Ex-4 than in those receiving saline (432.5 ± 36.1 µm^2^ vs. 319.3 ± 40.9 µm^2^, *n* = 17 for each group, *P* = 0.046) (Figure 1G and J). Immunohistochemistry staining showed that the numbers of α-SMA^+^ and CD68^+^; cells in the lesion decreased by 40% and 55%, respectively, in Ex-4-treated mice (α-SMA^+^: 31.3 ± 2.7 cells/µm^2^ vs. 18.8 ± 1.9 cells/µm^2^, *n* = 10 for each group, *P* = 0.001; CD68^+^: 32.8 ± 6.1 cells/µm^2^ vs. 14.8 ± 4.9 cells/µm^2^, *n* = 8 or 9, *P* = 0.03) (Figure 1H, I, K, and L).

### Ex-4 treatment inhibits HSPC proliferation in hypercholesterolemic LDLr^−/−^ mice

**Figure 2:**
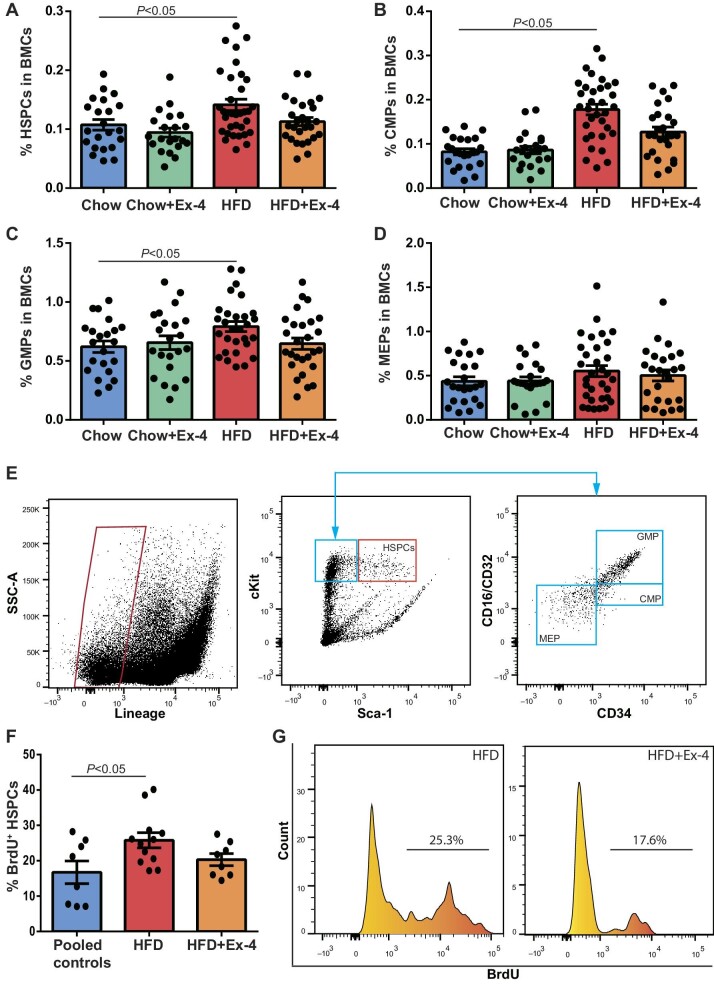
Ex-4 treatment inhibits HSPC proliferation in hypercholesterolemic LDLr^−/−^ mice. LDLr^−/−^ mice were placed on HFD for 12 weeks. After 6 weeks, mice received saline or Ex-4 treatment using minipump till the end of the experiment. (**A**–**D**) Frequencies of HSPCs, CMPs, GMPs, and MEPs in BMCs. *n* = 21–34. (**E**) A representative flow of FACS analysis for HSPCs, CMPs, GMPs, and MEPs. (**F**) Percentages of BrdU-incorporating HSPCs in the HSPC population. *n* = 8–12. (**G**) Representative FACS analysis for BrdU-incorporating HSPCs.

Freshly isolated BMCs were stained with HSPC markers for fluorescence-activated cell sorting (FACS) analysis. As shown in Figure 2A, administration of Ex-4 did not change HSPC frequency in LDLr^−/−^ mice on chow diet (chow: 0.11% ± 0.01%, chow + Ex-4: 0.09% ± 0.01%, *n* = 21 or 22, *P* = 0.28). HSPC frequency increased by 1.3-fold in mice on HFD, which was significantly decreased by Ex-4 treatment (0.14% ± 0.01% vs. 0.11% ± 0.01%, *n* = 27 or 34, *P* = 0.03). The frequencies of common myeloid progenitors (CMPs) and granulocyte–monocyte progenitors (GMPs) were 0.08% and 0.62% at baseline but increased to 0.18% and 0.79% by HFD, respectively (*P* < 0.05 for all), and were reduced in HFD-fed mice receiving Ex-4 (CMP: 0.18% ± 0.01% vs. 0.13% ± 0.01%, *n* = 26 or 33, *P* = 0.004; GMP: 0.79% ± 0.04% vs. 0.65% ± 0.05%, *n* = 27 or 29, *P* = 0.03) (Figure 2B and C). Nevertheless, megakaryocyte–erythroid progenitor (MEP) proportions were similar among all groups (*P* ≥ 0.18) (Figure 2D). Figure 2E shows a representative flow of FACS analysis for HSPCs, CMPs, GMPs, and MEPs.

To study whether Ex-4 treatment affected the cell cycle of HSPCs in mice, BMCs were labeled with BrdU and then stained with HSPC markers for FACS analysis. The percentages of BrdU-incorporating HSPCs in the HSPC population were 16.7%, 25.8%, and 20.3% in control, HFD, and HFD with Ex-4 groups, respectively (Figure 2F and G).

### GLP-1r expression in HSPCs

**Figure 3:**
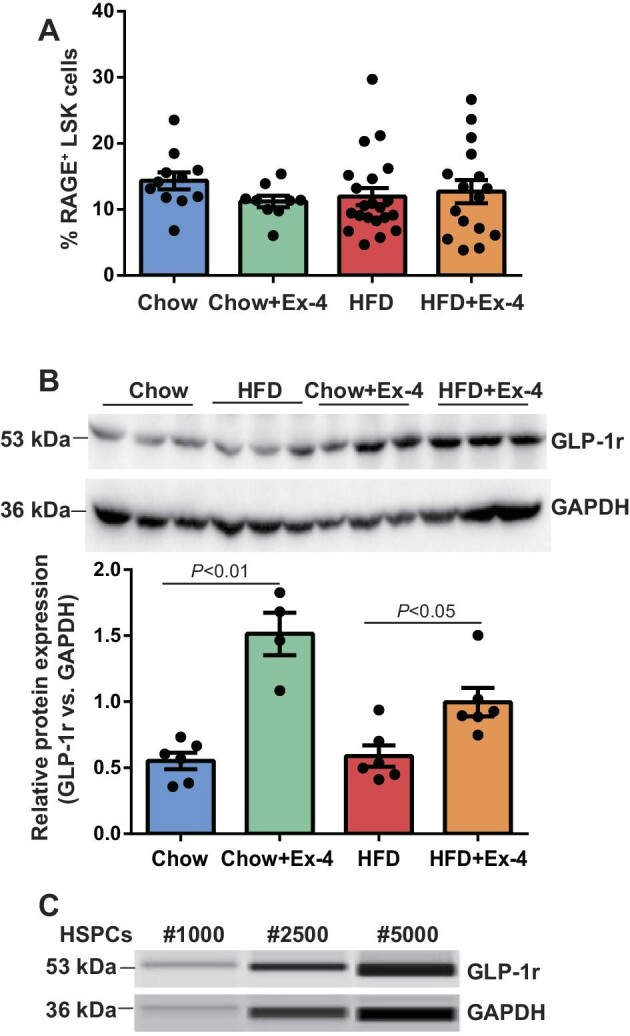
GLP-1r expression in HSPCs. LDLr^−/−^ mice were placed on HFD for 12 weeks. After 6 weeks, mice received saline or Ex-4 treatment using minipump till the end of the experiment. (**A**) BMCs were stained with HSPC surface markers and anti-mouse RAGE for FACS analysis. *n* = 9–21. (**B**) Western blotting for GLP-1r and GAPDH expression in livers. *n* = 4–6. (**C**) HSPCs were sorted out by FACS and subjected to capillary western blotting for GLP-1r and GAPDH expression. *n* = 3.

Previously, Nagareddy et al. (2013) reported that S100A8/S100A9 acted through the receptor for advanced glycation end-products (RAGE) on CMPs to promote granulocyte–macrophage colony-stimulating factor (GM-CSF) production that in turn stimulated GMP proliferation and myelopoiesis in type 1 diabetic mice and apoE^−/−^ mice. FACS analysis did not demonstrate a significant difference in the RAGE expression in HSPCs among groups (*n* = 9–21, *P* ≥ 0.09) (Figure 3A), while western blotting showed the increased GLP-1r expression in livers of Ex-4-treated mice (Figure 3B). Furthermore, wild-type HSPCs were sorted out by FACS, and GLP-1r protein expression was detected by capillary western blotting (Figure 3C).

### Phenotypes of GLP-1r^−/−^ mice

**Figure 4:**
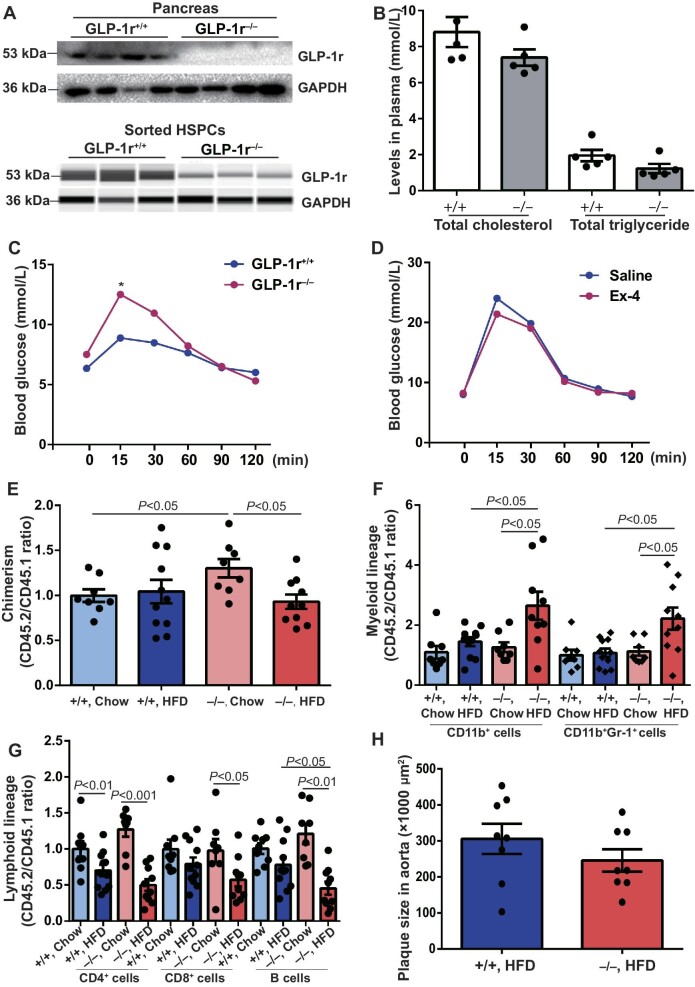
GLP-1r deficiency impaires glucose tolerance and white blood cell reconstitution. (**A**) Western blotting for GLP-1r expression in wild-type and GLP-1r^−/−^ pancreas and HSPCs. *n* = 4. (**B** and **C**) Lipid and glucose tolerance in wild-type and GLP-1r^−/−^ mice. *n* = 5–10. *P < 0.05. (**D**) Glucose tolerance in wild-type and GLP-1r^−/−^ mice on HFD with or without Ex-4 treatment. *n* = 9. (**E**–**H**) LDLr^−/−^ recipients were lethally irradiated and then injected with wild-type or GLP-1r^−/−^ BMCs. After 4 weeks, several recipients were switched to HFD. After 16 weeks, white blood cell chimerism, myeloid and lymphoid lineages, and plaque size were analyzed. *n* = 8–11.

To investigate the role of GLP-1r in HSPC biology, GLP-1r^−/−^ mice were established by the deletion of exon 5 and exon 6 using TALEN technology. Western blotting confirmed the deletion of GLP-1r in the pancreas and FACS-sorted HSPCs (Figure 4A). Fasting levels of plasma cholesterol and triglyceride were similar between wild-type controls and GLP-1r^−/−^ mice (Figure 4B). Remarkably, GLP-1r^−/−^ mice displayed impaired glucose tolerance when challenged with peritoneal injection of 10% glucose (Figure 4C). Ex-4 treatment did not improve glucose tolerance in GLP-1r^−/−^ mice fed on HFD, compared with saline (Figure 4D).

### BM reconstituting capacity of HSPCs in GLP1r^−/−^ mice

Thereafter, LDLr^−/−^ mice as recipients were lethally irradiated and then grafted with wild-type or GLP-1r^−/−^ BMCs, together with the equal amount of CD45.1 BMCs via tail vein. After 4 weeks, the recipients were fed on HFD for the following 12 weeks. After 16 weeks, FACS analysis showed that white blood cell chimerism in chow-fed LDLr^−/−^ recipients with GLP-1r^−/−^ BMC injection was 1.3-fold higher than that in chow-fed ones receiving wild-type BMCs, but was 1.4-fold lower than that in HFD-fed ones receiving GLP-1r^−/−^ BMCs (Figure 4E). Moreover, transplantation of GLP-1r^−/−^ BMCs, compared with transplantation of wild-type BMCs, led to increased myeloid cell production in LDLr^−/−^ recipients placed on HFD (Figure 4F). By contrast, B cell production was reduced in LDLr^−/−^ recipients receiving GLP1r^−/−^ BMCs, compared with receiving wild-type BMCs, when placed on HFD (Figure 4G). Nonetheless, plaque sizes were comparable between LDLr^−/−^ recipients injected with wild-type and GLP-1r^−/−^ BMCs and placed on HFD (Figure 4H). These results suggest the involvement of GLP-1r expression in HSPCs in lineage reconstitution.

### Ex-4 prohibits myeloid cell production induced by LDL through ABCA1-independent pathways

**Figure 5:**
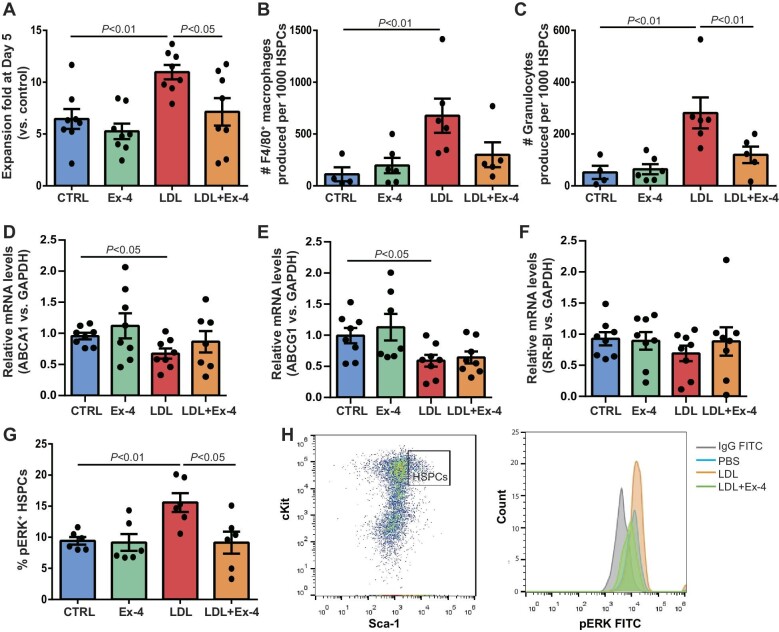
Ex-4 prohibits myeloid cell production induced by LDL through ABCA1-independent pathways. Wild-type HSPCs were sorted out by FACS and equal amounts of HSPCs were seeded in U-bottom 96-well plates. (**A**) After 5 days of culture in 0 or 100 µM LDL with or without 50 nM Ex-4, cells were harvested to calculate the expansion. *n* = 8. (**B** and **C**) The cells were stained with anti-mouse CD11b and anti-mouse Gr-1 for FACS analysis. *n* = 4–6. (**D**–**F**) Equal amounts of BMCs were cultured with LDL in the presence or absence of Ex-4 for 24 h. Cells were harvested to extract RNA for qPCR analysis of ABCA1, ABCG1, and SR-BI expression. *n* = 8 or 9. (**G**) BMCs were stimulated with LDL with or without Ex-4 for 5 min. Cells were stained with HSPC surface markers and pERK antibody for FACS acquisition. Percentage of pERK^+^ HSPCs in the HSPC population was analyzed by FACS. (**H**) Representative FACS analysis of pERK^+^ HSPCs. Left: HSPCs defined as cKit^+^Sca-1^+^ cells when gated on lineage^−/low^ cells. Right: pEKR^+^ cells when gated on HSPCs.

To test whether Ex-4 could regulate myeloid cell production, freshly isolated HSPCs were sorted by FACS and cultured *in vitro*. Immediately after seeding, cells were exposed to LDL-c at 0 or 100 µg/ml in the presence or absence of Ex-4. After 5 days, cell expansion was 6.5-fold at baseline but increased to 11.0-fold by LDL, which was abrogated by Ex-4 (*P* < 0.01 for LDL vs. baseline; *P* < 0.05 for LDL vs. LDL+Ex-4; *n* = 8) (Figure 5A). After 5 days of expansion, cells were harvested and stained with myeloid cell surface markers for FACS analysis. The numbers of F4/80^+^ macrophages and Gr-1^+^ granulocytes were increased by 11.0- and 7.1-fold in LDL-treated cells, respectively (*P* < 0.01 for LDL vs. baseline; *P* < 0.01 for LDL vs. LDL + Ex-4; *n* = 4–6) (Figure 5B and C). The addition of Ex-4 reduced granulocyte production by LDL (*P* < 0.05 for Gr-1^+^ granulocytes; *n* = 4–6) (Figure 5C).

Previous studies have consistently reported that hematopoietic deficiency of ABCA1 and SR-BI promoted skewed myelopoiesis and accelerated atherosclerosis. Here, BMCs were stimulated with 100 µg/ml LDL-c with or without Ex-4 for 24 h. Quantitative polymerase chain reaction (qPCR) analysis confirmed the significant reduction of ABCA1 and ABCG1 mRNA levels as well as a trend of reduction of SR-BI mRNA level in LDL-stimulated BMCs. However, there was no significant difference in the expression of these receptors between LDL-treated BMCs with and without Ex-4 (*P* ≥ 0.53) (Figure 5D–F). To test whether Ex-4 interfered ERK activation, wild-type BMCs were stimulated with phosphate-buffered saline (PBS) or 100 µg/ml LDL-c with or without 50 nM Ex-4 for 5 min. In line with the previous report, FACS analysis revealed that the proportion of pERK^+^ HSPCs in the HSPC population was 9.2% at baseline, increased to 15.0% after LDL stimulation, and declined to 10.0% in LDL-treated BMCs in the presence of Ex-4 (Figure 5G and H). These data suggest that ERK activation could be suppressed by Ex-4 in LDL-treated HSPCs.

### Metabolomics features of HSPCs by EX-4

**Figure 6:**
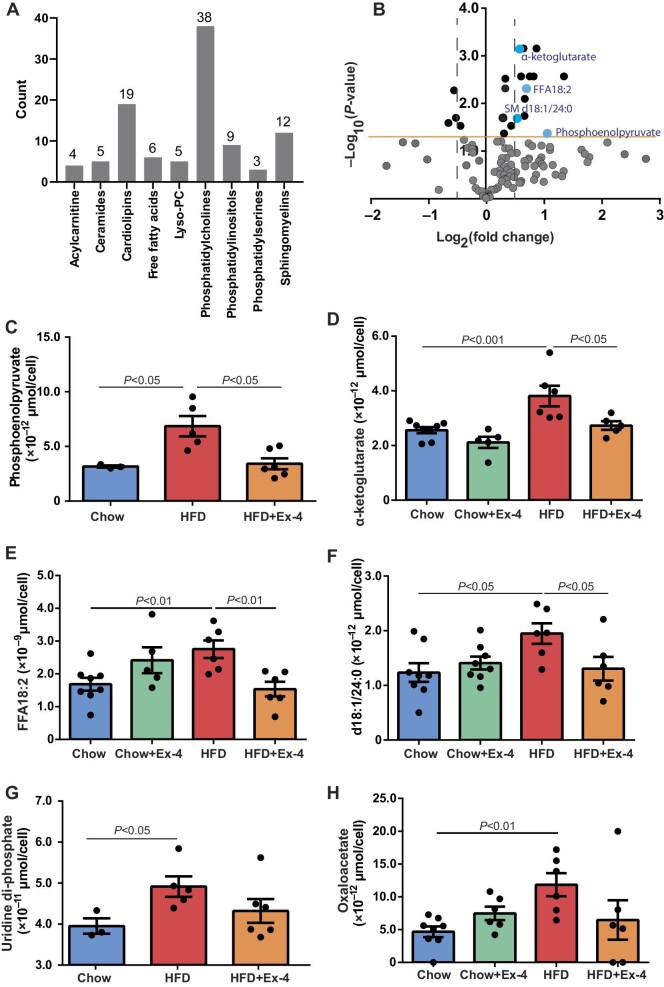
Metabolic profiles of HSPCs with Ex-4 treatment under HFD. LDLr^−/−^ mice were placed on HFD for 12 weeks. After 6 weeks, mice received saline or Ex-4 treatment using minipump till the end of the experiment. HSPCs were FACS-sorted for metabolomics measurement. (**A**) Lipidome summary. (**B**) Plot of differential metabolites between HSPCs of chow diet and HFD groups. Blue dots represent the metabolites with decreased levels in HSPCs of HFD-fed mice receiving Ex-4, compared with that of HFD-fed mice. (**C**–**H**) Intracellular levels of PEP, α-ketoglutarate, FFA18:2, SM d18:1/24:0, UDP, and oxaloacetate in HSPCs. *n* = 3–8.

To elucidate whether Ex-4 could modify metabolic profiles of HSPCs, LDLr^−/−^ mice were placed on chow diet or HFD for 12 weeks and received saline or Ex-4 treatment for the last 6 weeks. At the end of the experiments, HSPCs were sorted out for targeted metabolomics. In total, the intracellular levels of 119 metabolites, including 101 lipids from 9 lipid classes, 9 metabolites of the tricarboxylic acid (TCA) cycle, and 9 other metabolites, were quantified in HSPCs (Figure 6A). Among all, the levels of 21 metabolites were significantly different between HSPCs isolated from mice on chow diet and HFD (Figure 6B and Table 1). The results of the glycomics analysis were summarized in Supplementary Table S1.

**Table 1:** Differential metabolites in HSPCs among chow diet and HFD groups.

Metabolites	Chow diet	Chow + Ex-4	HFD	HFD + Ex-4	*P*-value
Number	3–8	3–8	5–6	4–6	
Metabolomics					
PEP (10^−12^ µmol/cell)	3.16 (3.01, 3.35)	NA	6.85 (5.02, 9.54)	3.42 (2.38, 5.06)*	0.005
UDP (10^−11^ µmol/cell)	3.95 (3.73, 4.33)	NA	4.92 (3.82, 5.62)	4.32 (3.82, 5.62)	0.04
α-ketoglutarate (10^−12^ µmol/cell)	2.56 (2.21, 2.91)	2.11 (1.75, 2.60)	3.81 (3.04, 5.40)	2.73 (2.41, 3.20)*	0.0007
Oxaloacetate (10^−12^ µmol/cell)	5.35 (3.62, 7.28)	7.48 (5.41, 10.80)	11.85 (7.58, 17.20)	9.72 (5.28, 20.81)	0.005
Lipidomics—phosphatidylcholines (PC)					
PC36:1 (10^−11^ µmol/cell)	4.20 (3.52, 5.12)	5.04 (3.52, 6.69)	6.32 (5.33, 7.30)	6.28 (5.77, 6.78)	0.001
PC36:2 (10^−10^ µmol/cell)	1.19 (0.98, 1.51)	1.71 (0.92, 2.44)	2.00 (1.55, 2.41)	1.93 (1.61, 2.23)	0.003
PC36:3 (10^−10^ µmol/cell)	1.14 (0.92, 1.28)	1.32 (0.77, 1.78)	1.52 (1.32, 1.73)	1.45 (1.22, 1.59)	0.03
PC38:2 (10^−12^ µmol/cell)	7.84 (7.18, 9.04)	12.46 (7.97, 17.73)	14.30 (13.15, 15.60)	14.73 (13.40, 15.98)	0.0007
PC38:3 (10^−11^ µmol/cell)	2.97 (2.71, 3.10)	4.10 (2.76, 5.59)	4.62 (4.09, 5.28)	4.94 (4.26, 5.47)	0.0007
PC38:4 (10^−11^ µmol/cell)	9.77 (8.89, 10.70)	11.64 (9.31, 14.43)	12.22 (10.98, 13.73)	13.17 (11.65, 14.30)	0.003
PC38:5 (10^−11^ µmol/cell)	6.89 (5.97, 7.76)	8.20 (5.94, 10.09)	8.39 (7.75, 9.26)	10.10 (9.16, 11.03)	0.02
PC40:3 (10^−12^ µmol/cell)	1.45 (1.15, 1.70)	2.02 (1.49, 2.64)	2.54 (1.92, 3.35)	2.06 (1.45, 2.62)	0.003
PC40:4 (10^−12^ µmol/cell)	4.13 (3.62, 4.82)	5.64 (4.42, 6.89)	6.25 (5.42, 6.99)	6.39 (5.21, 8.18)	0.003
PC40:5 (10^−11^ µmol/cell)	1.08 (1.02, 1.14)	1.29 (0.97, 1.64)	1.37 (1.18, 1.53)	1.59 (1.36, 1.79)	0.01
PC32:1e (10^−11^ µmol/cell)	2.14 (1.84, 2.49)	1.86 (1.45, 2.22)	1.56 (1.28, 1.90)	1.50 (1.26, 1.65)	0.03
PC34:1p (10^−11^ µmol/cell)	2.06 (1.64, 2.44)	2.34 (1.68, 2.93)	1.42 (1.19, 1.62)	1.40 (1.23, 1.58)	0.02
PC36:3p (10^−11^ µmol/cell)	3.71 (3.04, 4.49)	2.97 (2.43, 3.50)	2.51 (2.20, 2.88)	3.02 (2.69, 3.30)	0.01
Lipidomics—free fatty acid (FFA)					
FFA18:2 (10^−9^ µmol/cell)	1.68 (1.38, 1.96)	2.42 (2.10, 3.37)	2.76 (2.10, 3.37)	1.54 (1.13, 2.07)**	0.005
Lipidomics—sphingomyelins (SM)					
SM d18:1/18:1 (10^−12^ µmol/cell)	1.23 (0.95, 1.70)	1.41 (1.17, 1.64)	1.95 (1.52, 2.42)	1.31 (0.91, 1.71)	0.02
SM d18:1/22:1 (10^−12^ µmol/cell)	6.10 (4.93, 7.08)	6.38 (4.55, 8.37)	3.86 (2.96, 5.03)	3.99 (2.80, 5.01)	0.02
SM d18:1/24:0 (10^−11^ µmol/cell)	1.64 (1.35, 20.10)	2.31 (1.98, 2.55)	2.37 (2.06, 2.60)	1.66 (1.44, 1.89)*	0.02

Data were expressed as the mean with inter-quartile range. NA, not available. *P*-value indicates the difference of HSPCs between chow diet and HFD groups.

*P < 0.05 and **P < 0.01 for HFD vs. HFD + Ex-4.

Targeted metabolomics demonstrated that intracellular levels of phosphoenolpyruvate (PEP) and α-ketoglutarate were increased in HSPCs of HFD mice, both of which were decreased in HFD mice with Ex-4 treatment (Figure 6C and D). Of the 19 differential lipids, intracellular levels of one free fatty acid (FFA18:2) and one sphingomyelin (SM d18:1/24:0) were increased in HSPCs of HFD mice but decreased by Ex-4 treatment (Figure 6E and F). Compared with controls, intracellular levels of uridine di-phosphate (UDP) and oxaloacetate increased by 1.2- and 2.3-fold, respectively, in HSPCs of HFD mice but were not altered by Ex-4 treatment (Figure 6G and H).

**Figure 7:**
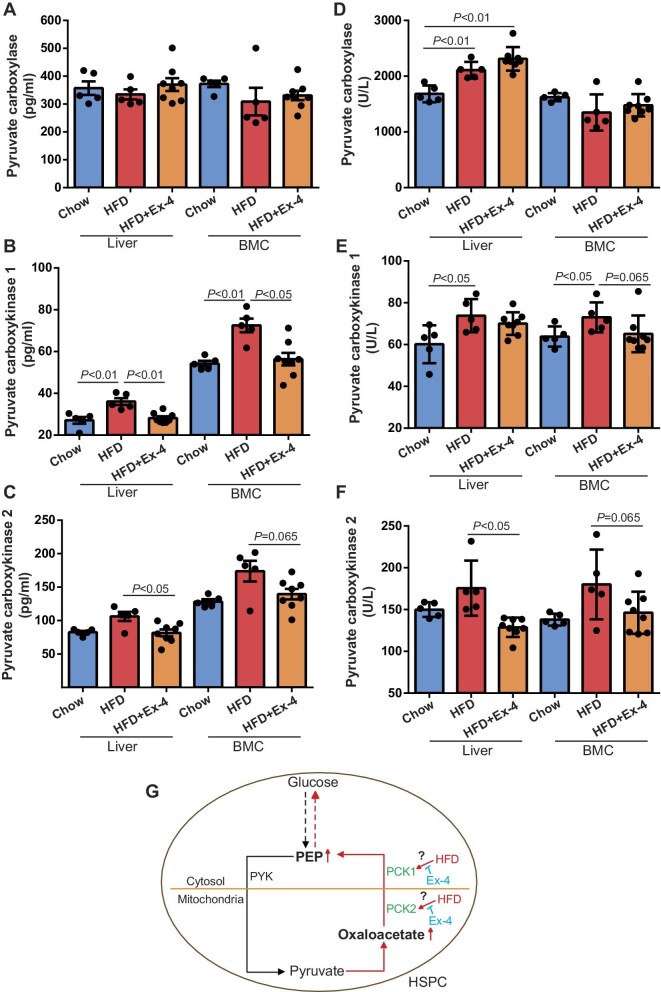
Protein expression and activity of enzymes related to PEP levels in the liver and BMCs of LDLr^−/−^ mice on HFD with or without Ex-4. Mice on chow diet were served as controls. (**A**–**F**) Proteins were extracted from the liver and BMCs of mice. Protein expression (**A**–**C**) and enzyme activity (**D**–**F**) of pyruvate carboxylase, PCK1, and PCK2 in the liver and BMCs were quantified by ELISA. *n* = 5–8. (**G**) A proposed model of glucolysis and gluconeogenesis in HSPCs of HFD mice with or without Ex-4 treatment. Black line and red line represent parts of the glycolysis and gluconeogenesis pathways in cells. PEP is the key metabolite linking glycolysis and gluconeogenesis. In glucolysis, pyruvate is generated from PEP by PYK. By pyruvate dehydrogenase, pyruvate can be converted to acetyl CoA to enter TCA cycle. Oxaloacetate is one of the metabolites in TCA cycle that could be metabolized to generate PEP by PCK1 and PCK2, which reside in the cytosol and the mitochondria, respectively. By targeted metabolomics, intracellular levels of PEP and oxaloacetate in HSPCs were found increased in HFD mice but decreased in HFD mice with Ex-4 treatment. Ex-4 treatment reduced PCK activity in HSPCs from HFD mice. Taken together, HFD enhances glucogenesis in HSPCs possibly via increasing PCK acitivity. Ex-4 circumventes gluconeogenesis in HSPCs of HFD mice by controlling PCK.

PEP is a key metabolite linking glycolysis and gluconeogenesis. To further dissect the metabolic features of HSPCs by Ex-4, the enzymes related to PEP production and catalysis were quantified by enzyme-linked immunosorbent assay (ELISA) in livers and BMCs. Hepatic pyruvate carboxylase levels were comparable in livers or BMCs among all groups (liver: *P* = 0.57; BMC: *P* = 0.48; *n* = 5–8) (Figure 7A). HFD enhanced pyruvate carboxykinase 1 (PCK1) expression by 1.2- and 1.3-fold in the liver and BMCs, respectively, which was abrogated by Ex-4 treatment (Figure 7B). Likewise, the expression of PCK2 was reduced in both the liver and BMCs of HFD mice receiving Ex-4, compared with that in HFD mice without Ex-4 treatment (Figure 7C).

Lastly, we determined the activity of these enzymes. Although pyruvate carboxylase expression levels were similar among all groups, the activities increased by 1.3- and 1.4-fold in the liver of HFD mice without and with Ex-4 treatment, respectively, compared with that in chow diet group (Figure 7D). Nonetheless, the activities of pyruvate carboxylase were similar in BMCs among all groups (*P* ≥ 0.18) (Figure 7D). Interestingly, a trend of reduction of PCK1 and PCK2 activities was observed in BMCs of HFD mice receiving Ex-4, compared with that in HFD mice without Ex-4 treatment (*P* = 0.065 for both) (Figure 7E and F). Taken together, these data demonstrate that Ex-4 treatment could alter the metabolism of HSPCs.

## Discussion

The proposed model of Ex-4 modulating HSPC glycolysis and gluconeogenesis is illustrated in Figure 7G, supported by several main findings of this study: (i) despite no changes in cholesterol and triglyceride levels, 6 weeks of Ex-4 treatment significantly retarded plaque progression in LDLr^−/−^ mice on HFD, which was associated with a reduced macrophage number in the lesion; (ii) GLP-1r expression was detected in HSPCs, and transplantation of GLP-1r^−/−^ BMCs resulted in skewed myelopoiesis in hypercholesterolemic LDLr^−/−^ recipients compared with those transplanted with wild-type BMCs; (iii) addition of Ex-4 to *in vitro-*cultivated purified HSPCs could antagonize LDL-induced HSPC expansion and inflammatory myeloid cell production; (iv) 6 weeks of Ex-4 treatment could modify the cellular metabolism of HSPCs (in the aspects of lipid metabolism, glycolysis, and TCA cycle) in hypercholesteremic LDLr^−/−^ mice, as evidenced by altered intracellular levels of PEP, α-ketoglutarate, FFA18:2, and SM d18:1/24:0; (V) PCK activity was lower in BMCs of Ex-4-treated HFD mice than that of HFD mice. Altogether, these data indicate that Ex-4 could suppress HSPC proliferation, myelopoiesis, and gluconeogenesis in HFD-fed LDLr^−/−^ mice.

Increasing body of evidence has illustrated the crucial link among hypercholesterolemia, HSPCs, skewed myelopoiesis, and atherosclerosis in mice and humans (Wang and Westerterp, 2020; Stiekema et al., 2021). Physically, GM-CSF and IL-3 are the key cytokines to promote myelopoiesis, both of which share common receptors located in the lipid raft in HSPCs. In the setting of hypercholesterolemia, deficiency of ABCA1, ABCG1, and apoE impairs cholesterol efflux in HSPCs and results in cholesterol accumulation in the lipid rafts, which promotes the recognition and activation of GM-CSF, IL-3, and pattern recognition receptors to enhance skewed myelopoiesis (Murphy et al., 2014; Ma and Feng, 2016; Gu et al., 2019; Stiekema et al., 2021).

Recently, Wong et al. (2022) reported that Ex-4 treatment dramatically reduced the production of a series of inflammatory cytokines in anti-CD3-treated wild-type mice (Stiekema et al., 2021). However, the anti-inflammatory effects of Ex-4 were diminished in mice with specific GLP-1r deletion in T lymphocytes (Wong et al., 2022), indicating that GLP-1 RAs could directly modulate immune cell function. Conversely, the inhibition of lipopolysaccharide-induced inflammation by Ex-4 was independent of the gut intraepithelial lymphocytes.

In this study, GLP-1r expression was detected in HSPCs by capillary western blotting (Figure 3C). Administration of Ex-4 attenuated HSPC proliferation and granulocyte production in LDLr^−/−^ mice on HFD and in HSPCs treated with LDL (Figures 1 and 5). However, Ex-4 treatment did not alter plasma cholesterol and triglyceride levels or the expression of ABCA1, ABCG1, and SR-BI receptors in HFD-fed LDLr^−/−^ mice (Figures 1 and 5), suggesting that the regulation of Ex-4 on HSPCs might be independent of these lipoprotein receptors. Interestingly, FACS data identified that ERK activation by LDL was suppressed by Ex-4 in HSPCs *in vitro* (Figure 5G and H). Previously, we reported that LDL triggered ERK phosphorylation in HSPCs and inhibition of ERK activation abolished granulocyte production by LDL *in vitro* (Feng et al., 2012). Thus, these data suggest that Ex-4 inhibits the ERK signaling pathway in HSPCs. Further studies are required to verify whether and how GLP-1 is implicated in the regulation of Ex-4 on HSPCs.

Metabolic regulation exerts a pivotal role in the maintenance of HSPCs (Karigane and Takubo, 2017). Taking the advantages of high-sensitivity targeted metabolomics and lipidomics, the intracellular levels of metabolites in limiting numbers of HSPCs were quantitated. PEP and α-ketoglutarate belong to the glycolytic and TCA cycles, respectively, both of which were increased in HSPCs of HFD mice but decreased in HSPCs of HFD-fed mice treated with Ex-4. PEP denotes a metabolic intermediate of glycolysis and is converted to pyruvate via pyruvate kinase (PYK), generating ATPs. Intracellular reduction in PEP indicates the abated aerobic glycolysis, which is critical to maintaining the progenitor function of HSPCs (Wang et al., 2014b). The reduction in α-ketoglutarate, a TCA cycle intermediate, implies an attenuated flux through the TCA cycle with Ex-4 treatment. Paradoxically, our data revealed that ATP production within TCA cycle was comparable in HSPCs between HFD and HFD plus Ex-4 groups, although Ex-4 treatment reduced blood glucose levels (Supplementary Table S1). Taken together, these data suggest that intermediate metabolites are involved in the regulation of HSPC proliferation by Ex-4.

Indeed, a growing body of evidence has demonstrated that metabolites and their related enzymes could modulate cell function in different manners. For instance, hepatic adenosine kinase overexpression elevated DNA methylation and reduced PPARα expression, leading to increased fatty acid oxidation and inflammation in non-alcoholic fatty liver disease (Feng et al., 2012). *In vitro*, the addition of PEP or sodium oxalate promoted cell cycle transition and mesenchymal stem cell proliferation via activation of Akt and STAT3 (Jeong et al., 2019). Untargeted metabolomics analyses uncovered the increase of 3-methyl-2-oxovalerate in endothelial cells activated by forkhead box O transcription factor (FOXO1), which promoted S-2-hydroxyglutarate production that, in turn, transcriptionally activated hypoxia-inducible factor-mediated cell cycle arrest, resulting in endothelial cell senescence (Andrade et al., 2021).

When dissecting lipidomics data, SM d18:1/24:0 and FFA18:2 in HSPCs were lower in HFD mice with Ex-4. Along this line, dietary supplementation with linoleic acids (FFA18:2) was previously reported to stimulate HSPC proliferation of transplanted BMCs in mice compared to non-supplemented donor mice (Limbkar et al., 2017). The precise nature of these metabolites in HSPC biology, however, needs further investigation.

Nevertheless, the study must be interpreted with limitations. First, differential levels of cellular metabolites were found in HSPCs of LDLr^−/−^ mice on HFD with or without Ex-4 treatment. Further studies are required to evaluate their effects on HSPCs *in vitro* and *in vivo*. Second, there are eight GLP-1 analogs that have been used in clinical practice, among which semaglutide is the latest one. It would be of interest to investigate whether all these analogs have different potencies for HSPC regulation. Third, the lipid-lowering effect of GLP-1a analogs has been reported in clinical trials of GLP-1 analogs (Lovshin et al., 2015; Gerstein et al., 2019). Paradoxically, the Ex-4 treatment did not alter plasma cholesterol and triglyceride levels in the study. It could be due to the high baseline levels of cholesterol and triglycerides induced by HFD. Fourth, HSPCs are tiny and quiescent population, which hampers the exploration of signaling pathways downstream of GLP-1/GLP-1r in these cells. Fifth, several FFA particles were detected increased in HSPCs isolated from HFD mice. Numerous studies have demonstrated that FFAs are implicated in oxidative stress and ROS production (Grandl and Wolfrum, 2018). Previously, we showed that inhibition of ROS production by N-acetyl-L-cysteine injection suppressed SR-BI^−/−^ HSPC expansion induced by HFD (Grandl and Wolfrum, 2018). We postulate that the altered lipids could enhance oxidative phosphorylation and ROS production, leading to skewed inflammatory myeloid cell production from HSPCs and accelerated atherosclerotic progress. However, the lipids were not available in the market. It is currently not feasible to investigate the effects of the lipids on HSPC number and function.

In conclusion, GLP-1r is expressed in HSPCs. Acting through GLP-1r, GLP-1 analogs directly regulate HSPC proliferation and myeloid cell production, resulting in the attenuation of inflammatory cell number and lesion size in the plaque of hypercholesterolemic LDLr^−/−^ mice.

## Materials and methods

### Mice and treatment

Male wild-type C57BL/6J mice, LDLr^−/−^ mice, and GLP-1r^−/−^ mice used in the study were all on CD45.2 (H-2 kb) background. B.6SJL-PTPRCA (CD45.1) mice were used as a competitor in the BM transplantation experiment. LDLr^−/−^ mice were purchased from GemPharmatech. GLP-1r^−/−^ mice were established by deletion of exon 5 and exon 6 via TALEN technology (ViewSolid Biotech). Briefly, a neomycin cassette replaced two exons of the gene, which encoded the first and third transmembrane domains and an intervening peptide sequence. Northern blotting and reverse transcription–PCR showed no detectable GLP-1r mRNA in the pancreas, lung, or hypothalamus of homozygous mutant mice. At the age of 12 weeks, LDLr^−/−^ mice were placed on HFD (1.25% cholesterol and 65% fat) (Wang et al., 2015). Meanwhile, mice were administrated with Ex-4 at 20 nM/kg/day via subcutaneously implanted osmotic pumps as described previously (Yoo et al., 2018).

All the mice were maintained in the animal facility in Beijing Youan Hospital, where they had free access to water and standard laboratory chow ad libitum. A total of 275 mice were used in the study. The protocols of mouse experiments were approved by the Ethics Committee of Capital Medical University.

### Competitive BM transplantation

At the age of 9 weeks, LDLr^−/−^ mice on chow diet were lethally irradiated at 9.0 Gy. After 6 h, BMCs were dissected from wild-type, GLP-1r^−/−^, and CD45.1 mice. After numeration, 5 × 10^6^ BMCs of wild-type or GLP-1r^−/−^ BMCs were mixed with 5 × 10^6^ CD45.1 BMCs and then injected into each irradiated LDLr^−/−^ recipient via tail vein. A total of 37 LDLr^−/−^ mice received BM transplantation, of which 19 mice were injected with wild-type BMCs and 18 mice were injected with GLP-1r^−/−^ BMCs. After 4 weeks, 11 LDLr^−/−^ recipients receiving wild-type BMC injection and 10 LDLr^−/−^ recipients receiving GLP-1r^−/−^ BMCs were switched from chow diet to HFD for 12 weeks. The rest of the recipients were maintained on chow diet till the end of the experiment.

Chimerism analysis was performed at 16 weeks after BM transplantation. Peripheral blood cells were harvested, red blood cells were lysed, and white blood cells were stained with anti-CD45.1 FITC, anti-CD11b PE, anti-CD45.2 PerCP-Cy5.5, and anti-Gr-1 APC antibodies to study myeloid lineage reconstitution by FACS. Peripheral blood cells were stained with anti-CD45.1 FITC, anti-CD45.2 PerCP-Cy5.5, and anti-B220 APC to evaluate B cell reconstitution. Cells stained with isotype control antibodies were used as the negative control for FACS analysis (BD, FACS Canto II). Afterwards, mice were sacrificed to dissect the heart for histological analysis.

### Lipoprotein preparation

LDL (1.006–1.063 g/ml) was isolated from human plasma samples by density gradient ultracentrifugation in a swing-out rotor as described previously (Jacobs et al., 2008). After overnight dialysis in PBS containing 1 mM EDTA, LDL was concentrated by an Amicon Ultra-4 50-kDa centrifugal filter (Millipore) at 4500 RPM (1100× *g*) for 8 min using a Sorvall Legend XF centrifuge (Thermo Fisher Scientific). LDL was collected and filtered through a 0.22-µm filter (Millipore). Cholesterol content in LDL (LDL-c) was quantified, aliquoted, and stored at −80°C.

### White blood cell counts

Leukocytes, lymphocytes, monocytes, granulocytes, red blood cells, and blood platelets in the peripheral blood were quantified with an Auto Hematology Analyzer (Mindray, BC-2800Vet).

### Glucose tolerance test and lipid profiles

Mice were fasted overnight. The next day, they were injected with 10% glucose (10 µl/g body weight) intraperitoneally. Blood glucose levels were determined before and at 15, 30, 60, 90, and 120 min after injection (Roche). Fasted blood samples were collected using EDTA as an anticoagulant. Triglyceride and cholesterol levels were determined using the triglyceride and cholesterol determination kits, respectively.

### ELISA

Proteins were extracted from the liver and BMCs of the mice, and 10 µg protein lysate was used to determine the enzyme concentrations of pyruvate carboxylase, PCK1, and PCK2 by ELISA, respectively (MLBio). In parallel, the enzyme activity was measured by ELISA (MLBio).

### Cell isolation and culture

Lineage^−/low^ cells were isolated from wild-type BMCs using the Lineage Cell Depletion Kit (Miltenyi Biotech) in accordance with the manufacturer's instructions. After staining with anti-lineage cocktail APC, anti-Sca-1 FITC, and anti-cKit PE antibodies, HSPCs were sorted on a FACS Aria III (Becton Dickinson). Equal amounts of BMCs and lineage^−/low^ cells were cultured in low-adsorption 24-well plates, and sorted HSPCs were seeded in U-bottom 96-well plates. Cells were cultivated in SFEM medium (STEMCELL Technologies) supplemented with 10 ng/ml stem cell factor (R&D), 10 ng/ml TPO (R&D), and 10 ng/ml IL-3 (R&D). On top of that, cells were exposed to PBS or 100 µg/ml LDL-c in the presence or absence of 50 nM Ex-4 (Abcam) for up to 5 days (Lee et al., 2012). After harvest, cells were counted and then stained with corresponding markers for FACS.

### FACS

BMCs were stained with surface markers of HSPCs, CMPs, GMPs, and MEPs for FACS analysis. HSPCs were defined as Lin^–^Sca-1^+^cKit^+^ cells (so called ‘LSK cells’); CMPs, GMPs, and MEPs were defined as CD34^+^CD16/32^dim^cKit^+^Sca-1^–^lineage^–/low^ cells, CD34^+^CD16/32^+^cKit^+^Sca-1^–^lineage^–/low^ cells, and CD34^–^CD16/32^dim^cKit^+^Sca-1^–^lineage^–/low^ cells, respectively (Larsen et al., 1989). To analyze the cell cycle of HSPCs, BrdU was injected peritoneally 12 h before sacrifice. In accordance to the manual, freshly isolated BMCs were stained with surface markers of HSPCs. After permeabilization and fixation, cells were stained with FITC-conjugated BrdU prior to FACS analysis.

To explore the signaling pathways downstream of Ex-4, freshly isolated BMCs (1 × 10^6^ cells/condition) were stimulated with PBS or 100 µg/ml LDL-c in the presence or absence of Ex-4 (50 nM) for 5 min. Cells were fixed and stained for HSPC markers. After that, cells were permeabilized by BD Cytofix/Cytoperm Fixation/Permeabilization Kit, following the manufacturer's manual. Then, cells were stained with rat anti-mouse phospho-p44/42 MAPK (Erk1/2) (Thr202/Tyr204) (1:50) for 30 min and consequently goat anti-rat IgG FITC (1:500) for 20 min prior to FACS analysis.

Isotype IgG antibodies were used as negative controls. To obtain sufficient cells for analysis, at least 100000 BMCs were acquired. Data were acquired by FACS Canto II (BD) and analyzed by FACSDiva (BD). FACS antibodies are listed in Supplementary Table S2.

### RNA extraction and quantitative analysis by qPCR

Total RNA was extracted using TRI reagent and converted to cDNA using the HiScript® III All-in-One RT SuperMix Perfect for qPCR kit. Real-time PCR was performed on the ViiA^TM^ 7 system (Applied Biosystems) using the Taq Pro Universal SYBR qPCR Master Mix reagent. qPCR primers are listed in Supplementary Table S3.

### Western blotting analysis

Mouse BMC extracts were prepared for western blotting analysis. Briefly, protein lysates (50 µg) were resolved on 10% SDS–PAGE gels, transferred onto polyvinylidene fluoride membranes, blocked with 5% (*w*/*v*) non-fat dried milk in Tris-buffered saline containing 0.05% Tween 20 for 1 h at room temperature, and then probed with the primary antibody overnight at 4°C. After washing, the membranes were incubated with horseradish peroxidase (HRP)-conjugated secondary antibodies for 1 h at room temperature, and protein bands were visualized by chemiluminescence. The primary antibodies used in this study were goat anti-rabbit GLP-1r (1:1000, NBP1-97308, NOVUS) and goat anti-rabbit GAPDH (1:1000, D16H11, Cell Signaling Technology).

### Capillary western blotting analysis

Equal amounts of FACS-sorted HSPCs were subjected to capillary western blotting for protein expression study as described previously (Zhang et al., 2019). Briefly, proteins were extracted, and the concentration was determined by BCA (Bio-Rad). Equal amounts of proteins were diluted with 0.1× sample buffer. Four parts of diluted samples and one portion of 5× Fluorescent Master mix were mixed and sucked up onto stacking gel. After blocking, goat anti-rabbit GLP-1r (1:1000, NBP1-97308, NOVUS) or goat anti-rabbit GAPDH (1:1000, D16H11, Cell Signaling Technology) was probed. HRP-conjugated secondary antibodies and chemiluminescent substrate were dispensed into designated wells in an assay plate. After plate loading, the separation electrophoresis and immunodetection steps took place in the fully automated capillary system.

### Histology

To investigate atherosclerotic lesions, heart tissues were fixed by 4% paraformaldehyde. Heart tissue sections (5 µm thick) were obtained from the paraffin block using a rotary microtome. By hematoxylin and eosin (H&E) staining, the severity of atherosclerotic lesions was evaluated by Image J (NIH).

### Immunohistochemistry

Paraffin sections were heated at 60°C for 2 h, dewaxed in xylene, and dehydrated through a gradient concentration of alcohol. After retrieving and blocking the endogenous peroxidase and non-specific staining with 3% H_2_O_2_ and 3% bovine serum albumin, the sections were incubated with donkey anti-mouse CD68 (1:200, ab201340, Abcam) or goat anti-rabbit anti-α-SMA (1:200, ab5694, Abcam) overnight at 4°C. The slides were then incubated with HRP-conjugated secondary antibody for 30 min at 37°C. Finally, the sections were visualized by diaminobenzidine solution and counterstained with hematoxylin.

### Targeted metabolomics and lipidomics analyses

Targeted omics analyses were conducted at LipidALL Technologies. Freshly sorted cells were inactivated in ice-cold chloroform:methanol (1:2, *v*/*v*). Lipids were extracted from cells using a modified version of Bligh and Dyer's protocol, as previously described (Lam et al., 2021). Lower lipid organic phase was dried in a SpeedVac under OH mode, and targeted lipidomics analyses were conducted on an Exion UPLC-coupled to Sciex 6500 Plus QTRAP. Internal standards used for lipid quantification included diC8-PI (Echelon Biosciences), d**_31_**-PS, LPC 17:0, DMPC, C12-SM, d**_3_**-16:0-carnitine, and Cer-d18:1/d**_7_**-15:0 from Avanti Polar Lipids, as well as FFA d**_31_**-16:0 from Sigma-Aldrich. The upper aqueous phase from Bligh and Dyer's extraction, containing polar metabolites, was dried under a stream of nitrogen and reconstituted in methanol:water (1:1). An aliquot of the polar metabolite extract was derivatized with 3-nitrophenylhydrazine for quantification of TCA cycle metabolites on a Thermofisher DGLC U3000 connected with Sciex 6500 Plus QTRAP as previously reported (Li et al., 2019). Internal standards included d**_4_**-citrate, d**_4_**-succinate, d**_3_**-pyruvate, d**_4_**-fumarate, and d**_3_**-malate from Cambridge Isotope Laboratories. Other polar metabolites were separated on a Phenomenex Luna Omega SUGAR column (i.d. = 100 × 2.1 mm, 3 µm) and quantitated under electrospray ionization mode using **^13^**C**_6_**-fructose-6-phosphate, **^13^**C**_6_**-glucose-6-phosphate, **^13^**C**_6_**-fructose-1,6-biphosphate, and **^13^**C**_6_**-UDP-glucose (Cambridge Isotope Laboratories) as internal standards.

### Statistical analyses

Data were expressed as mean ± standard error of the mean (SEM). Statistics analysis was performed using GraphPad Prism (GraphPad Software Inc.). For the comparison between two experimental groups, the unpaired, two-tailed Student's *t*-test was used for the data with normal distribution; otherwise, the non-parametric Mann–Whitney analysis was used. For the comparison among three or more groups, one-way analysis of variance (ANOVA) with Dunnett was used when comparing treated groups against control, whereas ANOVA with Bonferroni was applied when comparing all groups. *P* < 0.05 was considered significant.

## Supplementary Material

mjad014_Supplemental_File
