# Supplementary material for: Exendin-4 attenuates atherosclerosis progression via controlling hematopoietic stem/progenitor cell proliferation
Source: J Mol Cell Biol. 2023 Mar 2;15(2):mjad014. doi: 10.1093/jmcb/mjad014 (PMC10478625; doi:10.1093/jmcb/mjad014)
Supplement: mjad014_Supplemental_File [file mjad014_supplemental_file.pdf]

Supplementary material

Exendin-4 attenuates atherosclerosis progression via controlling hematopoietic stem/progenitor cell proliferation

Cen Yan[1,†], Xiaojuan Ma[2,†], Sin Man Lam[3,†], Yuejie Zhang[1], Yu Cao[1], Yuan Dong[1], Li Su[4], Guanghou Shui[3,*], and Yingmei Feng[1,*]

[1] Department of Science and Development, Beijing Youan hospital, Capital Medical University, Beijing 100069, China

[2] Center of Basic Medical Research, Institute of Medical Innovation and Research, Peking University Third Hospital, 49 North Garden Road, Haidian District, Beijing, 100191, China

[3] State Key Laboratory of Molecular Developmental Biology, Institute of Genetics and Developmental Biology, Chinese Academy of Sciences, Beijing 100101, China

[4] Neuroscience Research Institute, Peking University Center of Medical and Health Analysis, Peking University, Beijing 100191, China

[†] These authors contributed equally to this work.

[*] Correspondence to: Ying-Mei Feng, E-mail: yingmeif13@sina.com, yingmeif13@ccmu.edu.cn;
Guanghou Shui, E-mail: ghshui@genetics.ac.cn

Supplementary Table S1 Summary of glycomics analysis in HSPCs.

	**Chow**	**HFD**	**HFD+Ex-4**	***P*-value**
Number	3	5	6	
Fructose-6-phosphate (10^{-13} μmol/cell)	7.14 (6.44, 8.24)	9.50 (7.83, 11.6)	8.88 (6.90, 10.98)	0.10
Glucose-6-phosogate (10^{-10} μmol/cell)	1.71 (1.54, 1.97)	1.93 (1.56, 2.33)	1.64 (1.52, 1.74)	0.50
Ribose-5-phosphate (10^{-11} μmol/cell)	9.64 (9.16, 10.00)	10.61 (9.73, 11.45)	9.64 (8.96, 10.20)	0.15
Fructose-1,6-bisphosphate (10^{-13} μmol/cell)	4.05 (3.59, 4.32)	4.10 (3.50, 4.59)	3.76 (3.47, 4.13)	0.91
Phosphoenolpyruvate (10^{-12} μmol/cell)	3.16 (3.01, 3.35)	6.85 (5.02, 9.54)	3.42 (2.38, 5.06)*	0.005
Uridine diphosphate-glucose (10^{-13} μmol/cell)	1.36 (1.19, 1.71)	1.35 (1.04, 1.62)	1.30 (1.22, 1.42)	0.94
Uridine diphosphate (10^{-11} μmol/cell)	3.95 (3.73, 4.33)	4.92 (4.49, 5.39)	4.32 (3.82, 4.77)	0.04
Adenosine monophosphate (10^{-12} μmol/cell)	2.13 (2.03, 2.22)	2.28 (1.91, 2.67)	2.00 (1.78, 2.21)	0.56
Adenosine triphosphate (10^{-12} μmol/cell)	1.41 (1.21, 1.53)	1.60 (1.40, 1.80)	1.50 (1.23, 1.82)	0.23

Data were expressed as mean with inter-quartile range. *P*-value indicates the difference of HSPCs between Chow and HFD groups. *$P < 0.05$ for HFD vs. HFD+Ex-4.

Supplementary Table S2 FACS antibodies.

Antibodies	Clones/catalogue number	Company
Anti-mouse Sca-1 PerCP-Cy5.5, FITC	D7	eBioscience
Anti-mouse Sca-1 PE-cy7	D7	BD
Anti-mouse cKit PE	2B8	eBioscience
Anti-mouse cKit APC-H7	2B8	BD
Anti-mouse lineage cocktail APC	M1/70, 145-2C11, RB6-8C5, TER-119, RA3-6B2	BD
Anti-mouse CD16/32 PercP-cy5.5	2.4G2	BD
Anti-mouse CD34 APC-eFluor 700	RAM34	eBioscience
Anti-mouse BrdU FITC	/	BD
Anti-mouse CD11b FITC	M1/70	BD
Anti-mouse Gr-1 APC	RB6-8C5	BD
Anti-mouse CD45.1 FITC	A20	BD
Anti-mouse CD45.2 PerCP-Cy5.5	104	BD
Anti-mouse CD45R/B220 APC	Ly-5	BD
Anti-mouse CD4 FITC	GK1.5	eBioscience
Anti-mouse CD8 PercP-Cy5.5	53-6.7	eBioscience
Phospho-p44/42 MAPK (Erk1/2)	D13.14.4E	Cell Signaling Technology

Supplementary Table S3 Sequences of the primers used for qPCR.

Gene	Forward	Reverse
SR-BI	TTTGGAGTGGTAGTAAAAAGG	TGACATCAGGGACTCAGAGTAG
ABCA1	TAGCAGCACCGTGTCTTGTC	TACGGCAGCACATAGGTCAG
ABCG1	CTTTCCTACTCTGTACCCGAGG	CGGGGCATTCCATTGATAAGG
GAPDH	AGGTCGGTGTGAACGGATTTG	TGTAGACCATGTAGTTGAGTCA
